# Evidence of Spreading Zika Virus Infection Caused by Males of Different Species

**DOI:** 10.3390/v14092047

**Published:** 2022-09-15

**Authors:** Thayane da Encarnação Sá-Guimarães, Monica Ferreira Moreira

**Affiliations:** Department of Biochemistry, Institute of Chemistry, Universidade Federal do Rio de Janeiro, Rio de Janeiro 21941-909, Brazil

**Keywords:** Zika virus, reproductive tract, males, *Aedes aegypti*

## Abstract

Zika virus (ZIKV) is a positive-sense single-stranded RNA flavivirus and is mainly transmitted by Aedes mosquitoes. This arbovirus has had a significant impact on health in recent years by causing malformations, such as microcephaly in babies and Guillain–Barré syndrome in adults. Some evidence indicates that ZIKV can be sexually transmitted and may persist in the male reproductive tract for an extended period in humans. Knockout and vasectomized mice have been used as models to reveal ZIKV infection in the male reproductive tract as a virus source. ZIKV presence in male and female mosquito reproductive tracts and eggs point to venereal and vertical/transovarian transmission, again demonstrating that the reproductive tract can be involved in the spread of ZIKV. Moreover, eggs protected by eggshells have the potential to be a ZIKV reservoir. Given the +-lack of vaccines and therapies for Zika fever and the underestimated prevalence rate, an understanding of ZIKV infection and its spread from the reproductive tract, which is protected from the immune system and potentially active for virus transmission, is imperative. We must also develop cheaper, more efficient techniques for virological surveillance inside vectors and humans, control vectors with ecofriendly insecticides, and promote condom use to avoid ZIKV contamination during sexual intercourse, as recommended by the World Health Organization.

## 1. Contextualization (What Is ZIKV?)

Zika fever is a disease caused by Zika virus (ZIKV), which is a positive single-stranded RNA arbovirus belonging to the Flaviviridae family that is mainly transmitted through the bite of infected *Aedes aegypti* or *Aedes albopictus* mosquitoes. Nonvector horizontal transmissions, such as blood transfusion and organ transplantation, vertical (mother to fetus) transmission and sexual (vaginal, anal and oral) transmission, have also been reported [1,2].

Until 2015 and 2016, ZIKV was only associated with self-limiting symptoms, similar to those of dengue fever. In the recent epidemic period, in Brazil, ZIKV was also associated with an increase in severe disease cases, such as microcephaly in newborns, and Guillain–Barré syndrome in adults [3,4,5]. Reports of similar cases of microcephaly were also linked, retrospectively, in French Polynesia in 2014 [6,7].

The majority of patients, ca. 80%, are asymptomatic [8]. The symptoms observed include rash, nonpurulent conjunctivitis, fever and arthralgia; however, in some cases, attention is called to the presence of the virus in the male genital tract that causes hematospermia, prostatitis and dysuria [8,9,10].

## 2. Reports of Sexual ZIKV Transmission in Humans

Sexual transmission was reported for the first time in 2011, with one case describing a man who, on returning to Colorado (USA) from a journey to Senegal in 2008, had unprotected sex with his partner while he showed arbovirus symptoms. In a few days, his partner showed similar symptoms, and ZIKV diagnosis was confirmed by serological methods. The sexual transmission was recognized due to a Zika vector absence in Colorado State [9]. Another case occurred in 2014: an Italian couple had symptoms consistent with ZIKV infection after the man’s vacation in Thailand. Their Zika diagnosis was confirmed only in 2016 by a plaque reduction neutralization test (PRNT), signaling possible sexual transmission [11]. In the same year, in Germany, an index patient returning from Puerto Rico had a confirmed ZIKV diagnosis; he had sexual intercourse with his partner, who also had a positive diagnosis for ZIKV, confirmed by RT–PCR. It is important to highlight that in the area where the couple lived, there was no description of virus–vector circulation [12]. There was also at least one case in which the index patient was asymptomatic; he was able to transmit the virus to his partner through sexual contact [13].

Another pattern of ZIKV transmission was observed for oral sex. A female resident in France, without a recent history of traveling to endemic sites, presented symptoms of Zika fever for approximately 7 days in 2016. She reported unprotected vaginal sexual intercourse (without ejaculation) and oral intercourse (with ejaculation) with a partner who had traveled to Brazil. The virus sequences obtained from both the man and woman had only four synonymous mutations between them, which corroborates the hypothesis of sexual transmission [14]. One of the longest periods of sexual transmission described in the literature occurred in 2016, when a woman showing symptoms between 32 and 41 days after the onset of symptoms from the index case (male–female) was later considered a case of sexual transmission, with an incubation period between 3 and 12 days [15]. Several studies reported the occurrence of cases with characteristic symptoms of ZIKV infection through possible sexual transmission (male–female) in which the index patients lived in countries without a description of the vector’s circulation (mosquitoes of the genus *Aedes*) but had been in endemic regions for some time, and during the trip or when returning the patients presented symptoms with the confirmation of a ZIKV infection by molecular diagnosis or serological methods [16,17,18,19].

There was only one suspected case of female-to-male sexual ZIKV transmission: a nonpregnant woman in her twenties infected her partner on Day 0 after travelling to an area with ZIKV transmission where she engaged in a single event of condomless vaginal intercourse with her partner. She developed several symptoms, and on Day 3, ZIKV RNA was detected in her blood and urine specimens. Although the serum testing for anti-Zika virus immunoglobulin M (IgM) was negative seven days after sexual intercourse (Day 6), the woman’s male partner also presented symptoms, and on Day 9, ZIKV was detected in his urine but not serum by an RT–PCR assay. Zika virus IgM antibodies were not detectable [20]. The prevalence of unprotected sex among men and women infected with ZIKV can contribute to the spread of the disease, indicating the need for men and women to use condoms.

## 3. Detection of ZIKV in Semen—RNA and Isolation

To confirm sexual transmission, studies have been performed to investigate ZIKV viral RNA in semen. Some studies were able to detect the virus within 7 days of the onset of symptoms [21,22] and other studies were able to detect the virus in the later stages of infection [14,18,23] at 18 [14], 31 days [24], 45 days [12], 76 days [16] and 92 days [25]. For other cases, later detections were performed 134 days [26], 158 days [27], 160 days [21] and 370 days after the initial infection, with longer periods described in healthy and symptomatic patients [28].

In addition, some studies call for attention to the detection of viral ZIKV RNA in semen in unexpected circumstances, such as vasectomized, immunosuppressed and asymptomatic men. Musso et al. [22] and García-Bujalance et al. [13] detected the presence of ZIKV RNA in the semen of asymptomatic patients 54 and 68 days after infection, respectively. Froeschl et al. [29] and Mead et al. [30] detected ZIKV RNA in the semen of vasectomized patients for up to 77 and 281 days, respectively. The longest period of time reported in the literature for the detection of ZIKV viral RNA in semen was 515 days in an immunosuppressed and asymptomatic patient [31].

The presence of RNA is not synonymous with an infectious viral particle, seeing that few studies have actually managed to isolate infectious ZIKV in cell cultures from semen samples collected soon after the onset of symptoms after 13 days [32], and later at 69 days [23]. The longest period described for isolating infectious particles was after 117 days [27]. This highlights the study of Froeschl et al. [29], who managed to isolate ZIKV from a sample of vasectomized patients 21 days after the onset of symptoms. Other studies have also been successful in isolating infectious viral particles from semen samples [14,24,33,34]. Aggregated data on the detection of ZIKV RNA from 37 case reports indicate a median duration of the detection of ZIKV of 40 days (95% CI: 30–49 days), and a maximum duration of 370 days in semen [35]. In a recent study, fifteen men had an extended follow up, three of whom were positive for Zika virus RNA in semen after 148 days and 150 days, and a third man was positive for Zika virus RNA after 414 days, the longest reported duration of Zika virus shedding in semen for a nonimmunosuppressed patient. However, this semen sample was not capable of infecting Vero E6 cells, reflecting either a nonreplicative virus in this cell or insufficient replicative viral shedding [36]. For women, at least two studies were able to detect the presence of ZIKV RNA in vaginal secretions. Reyes et al. [37] detected this in samples from 60 to 180 days after the onset of symptoms, and Sánchez-Montalvá et al. [38] used a 37-day vaginal swab.

## 4. Analysis of Semen

For ZIKV analysis, semen samples were separated into total, seminal plasma and cell fractions to investigate the presence of viral RNA or infectious particles. Biava et al. [39] detected viral RNA in the semen cell fraction, which could be indicative of the presence of infectious particles of ZIKV in these cells. Joguet et al. [21] observed characteristics of semen over time after ZIKV infection. At 30 days post infection (dpi), there was an increase in multiple anomalies, such as total sperm count, motility, vitality, and morphology, and in the titers of reproductive hormones, maintained at 90 dpi, with the recovery of sperm counts at 120 dpi. The authors suggest that more studies are necessary to prove that semen alterations after acute ZIKV infection might affect the fertility and patterns of the testes and epididymis. Older men and those with less frequent ejaculations have a greater tendency for prolonged ZIKV shedding than younger men with a higher frequency of ejaculation [30]. Avelino-Silva et al. [10], despite methodological limitations and a restricted number of patients, reported sperm parameters for one year after Zika virus infection that included abnormal sperm morphology in four of the six men analyzed suggesting a possible persistent harmful effect on male fertility caused by acute ZIKV infection in humans detectable 12 months after infection, as spermatogenesis takes three months. Borges et al. [40] stated that the clinical implications of ZIKV compartmentalization in whole semen need to be better investigated. However, this is a matter of concern because ZIKV seems to be the only arbovirus that causes such severe symptoms of infertility in humans.

## 5. Experiments with Mice

Studies in vivo have been performed to try to understand how to study ZIKV infection, using groups of immunocompetent and immunodeficient mice as a model [41,42]. These studies with specific immunodeficient mice (e.g., *Ifnar^-^*, AG129: knockout for the receptor of interferon α (IFN-α), β (IFN-β) and γ (IFN-γ)) have shown effects on the reproductive tissues of males, such as mild orchitis and acute inflammation. A mixed inflammation frame was observed in the epididymis and testicles when infected with ZIKV. In addition, some Leydig cells showed immunoreactivity to the ZIKV antigen. In another study, male adult wild-type mice (e.g., C57BL/6) were treated with anti-IFN-α and IFN-β receptor 1 (*Ifnar1*) antibodies, and the accumulation of particles or viral RNA alterations was observed in the testicles and epididymis, as shown in mice infected with ZIKV. These parameters were also evaluated in mature sperm when compared to the result of the controlled mice (isotype control antibody) that had no accumulation of particles or viral RNA alterations in the testicles [43].

In studies using the immunocompetent mouse model, the infectious virus was detected in the ejaculates of vasectomized and nonvasectomized AG129 mice between 7 and 21 dpi, and viral RNA was detected up to 58 dpi. The fluids of vasectomized animals had significantly lower viral titers, and the infectivity interval was shorter [41]. Mcdonald et al. [44] also used male AG129 wild-type mice and detected viral infections in the testes, epididymis, and seminal vesicles after inoculation with different ZIKV strains (PRVABC59, P6-740 or FSS 13025). Other effects, such as splenomegaly, declining sperm counts and increased testicular weight, were observed in ZIKV-infected animals. ZIKV RNA was detected mainly in the epididymis, but was also located in the seminiferous testis and tubules.

Using four strains of ZIKV (Asian genotype ZIKV strains: PRVABC59—Puerto Rico, 2015; FSS13025—Cambodia, 2010; P6-740—Malaysia, 1966. African genotype ZIKV strain: DakAr41524—Senegal, 1984), McDonald, Duggal and Brault [45] assayed the relative efficiency of sexual transmission of these ZIKV strains through subcutaneous and intraperitoneal inoculation routes. The potential capacity to be sexually transmitted and viral loads in the male reproductive tract and in seminal fluids were assessed in IFN-α, IFN-β and IFN-γ receptor-deficient (AG129) mice; there was no difference between the African and Asian genotypes in sexual transmission rates in AG129 mice, indicating that the adaptation of the virus would hardly have caused the American outbreak. In females, the intravaginal inoculation of ZIKV was able to cause infection. However, pretreatment with progesterone was essential to increase the mortality of female mice [41,42]. Another study with AG129 females showed that the inoculation route interferes with the viral titer, with the sexual route capable of generating higher viral titers when compared to the others (intravaginal and subcutaneous), regardless of pregnant or nonpregnant status, indicating that a change in tropism and the spread of the virus may occur in the female reproductive tract [46].

ZIKV strains with nonsynonymous variations in some residues of proteins E and NS1 caused damage in tissues, such as the brain and epididymis [47]. Using chimeric strains in the structural genes prM/E, Mcdonald et al. [48] determined that these genes were determinants in viremia but did not influence sexual transmission, and Fulton et al. [49] observed that even with the degree of flexibility of protein E, it did not suffer from antigenic drift.

## 6. Virus Replication Sites in the Human Male and Animal Reproductive Tracts

In their study, Froeschl et al. [29] described that in a patient who was successfully vasectomized, spermatocytes could be excluded as carriers of viral particles. However, the authors suggest that the Cowper’s glands, prostate, seminal vesicles, and peroxidase-positive round cells or inflammatory cells (e.g., neutrophils, lymphocytes, macrophages, and epithelial cells) found in ejaculate are possible sources of ZIKV replication in the male reproductive tract. Infectious ZIKV was detected by Govero et al. [43] in mature sperm from mice treated with anti-IFNα antibody and IFNβ receptor 1 (*Ifnar1*), and by RNA in situ hybridization (ISH) of spermatogonia, primary spermatocytes, trophic Sertoli cells, mature sperm cells, and Leydig cells. However, they noted that in the regions with the highest amount of ZIKV RNA, a large increase in leukocytes and tissue damage was observed, suggesting inflammation formation. Huits et al. [50] assumed that the virus replicates in seminiferous epithelial cells, with the average duration of virus detection in semen coinciding with the sperm production and maturation cycle (≈74 days). One possible way to access immunoprivileged sites, such as the testicles (cross the blood–testis barrier—BTB) and the lumen of the epididymis would be through infected leukocytes. Infectious particles could be found in association with seminal fluid cells or free [44].

## 7. Mosquitoes

The practice of hematophagy by females of *A. aegypti* is essential to the egg production process, which is initiated by the neuroendocrine system to begin vitellogenesis and the gonotrophic cycle [51]. The eggshell generated will consist, in part, of a chitin-like component that makes them extremely resistant to environmental conditions [52,53] and might be useful to protect viruses, such as ZIKV, inside eggs. Accordingly, ZIKV was detected in the ovaries of *A. aegypti* infected by oral feeding in vitro by electron microscopy and real-time PCR techniques [54].

In addition, in the studies by Ciota et al. [55] with field mosquitoes and Li et al. [56] with *A. aegypti* from colonies fed with infectious blood, it was observed that these mosquitoes were able to transmit ZIKV to their offspring, signaling the transovarian transmission capacity of this insect for ZIKV. In addition, the production of viable eggs was not significantly altered, even when the female *A. aegypti* was infected, which makes these eggs theoretically a source of viral maintenance in the environment [57,58]. The presence of ZIKV in male and female mosquitoes and in their eggs points to venereal and vertical/transovarian transmission. Therefore, a collection of male and female *Aedes aegypti* from the field naturally infected with ZIKV in Brazil, made by Ferreira-de-Brito et al. [59], suggests vertical and/or venereal transmission in mosquitoes. In addition, males and females of *A. aegypti* and *Aedes albopictus* collected in Medellín during the post-epidemic period of 2017 and 2018 were positive for ZIKV, evidencing the role mosquitoes have in maintaining ZIKV during inter-epidemic periods in nature, with *A. albopictus* acting as a secondary vector for this flavivirus [60]. Likewise, Ciota et al. [55] collected mosquitoes in Argentina, Mexico and New York (USA), and had positive results when detecting ZIKV in their offspring. Venereal transmission was successfully reproduced in laboratory assays when ZIKV RNA was detected in the sexual fluids of both sexes of *A. aegypti*, after mating artificially infected mosquitoes with uninfected mosquitoes [61,62]. Venereal transmission in mosquitoes was confirmed when the sequenced genomes of ZIKV from venereal and oral routes were compared. The results showed that there were no significant genetic differences in the genomes recovered from venereal-infected mosquitoes [61]. The offspring of the first gonotrophic cycle of *A. aegypti* females that were fed blood infected with ZIKV were successfully detected, and transovarian transmission in these mosquitoes was suggested [56]. Although, *A. aegypti* vectorial capacity is affected by the route of infection and it also reduces lifespan [63]. Padilha et al. [58] and Silveira et al. [63] observed, independent of the virus strain, that ZIKV infection does not affect the egg production of *A. aegypti* females, neither in quantity nor in viability.

## 8. Conclusions

In general, the data collected in this review indicate that the presence of ZIKV in the male reproductive tract of several species (for example, humans, mice and mosquitoes) shows that this is a possible site of viral reproduction, protected from the immune system, having real potential for the transmission of Zika virus. This makes it imperative to develop cheaper and more efficient techniques for the surveillance of ZIKV and Zika disease. For humans, the CDC [64] recommends abstaining from unprotected sex for a period of up to 3 months, while the WHO [2] recommends 6 months for men and 2 months for women, and also recommends men and women delay plans to have children if they have traveled to regions endemic for the Zika virus, so as to avoid possible adverse pregnancy outcomes. Therefore, as suggested by Matheron et al. [65] and reviewed by Petridou et al. [31], the time oriented toward sexual abstention may be insufficient and should be reviewed and calculated on a case-by-case basis, especially for immunosuppressed people. Without a safe vaccine and without adequate therapy, it is essential to monitor and control vectors as a strategy to avoid the disease. Thus, challenges remain in developing new ecofriendly insecticides, and other insect control methods, and developing methods for viral detection within vectors and within humans to prevent the spread of Zika fever.

## Data Availability

Not applicable.
